# Comprehensive analysis of the clinical manifestations and hematological parameters associated with secondary immune thrombocytopenia in patients with primary Sjögren syndrome: An observational study

**DOI:** 10.1097/MD.0000000000037909

**Published:** 2024-05-10

**Authors:** Wenwen Yang

**Affiliations:** a Department of Medical Nursing, Cangzhou Medical College, Cangzhou, Hebei Province, China.

**Keywords:** hematological parameters, logistic regression analysis, primary Sjögren syndrome, secondary immune thrombocytopenia

## Abstract

Primary Sjögren Syndrome (pSS) is a chronic autoimmune disease that primarily affects exocrine glands and can lead to various extraglandular manifestations, including secondary immune thrombocytopenia (ITP). Understanding the clinical and hematological differences in pSS patients with and without secondary ITP is crucial for improved patient management and treatment strategies. This retrospective study, conducted from January 2020 to December 2023, involved a cohort of pSS patients, dividing them into 2 groups: those with secondary ITP and those without. Patients were evaluated using the European League Against Rheumatism Sjögren Syndrome Disease Activity Index (ESSDAI), EULAR Sjögren Syndrome Patient-Reported Index (ESSPRI), Health Assessment Questionnaire, and other hematological parameters. Inclusion criteria were based on the American-European Consensus Group or ACR/EULAR classification criteria for pSS. Exclusion criteria included other autoimmune or hematological disorders, prior splenectomy, recent blood transfusions, and lack of informed consent. Statistical analysis was performed using SPSS software, with various tests applied to analyze the data, including logistic regression to identify risk factors for secondary ITP. Significant differences were noted in fatigue, lymphadenopathy, arthritis, mean age, and ESSDAI scores between the secondary ITP and non-secondary ITP groups. Patients with secondary ITP exhibited higher platelet counts, more prevalent lymphopenia, higher immunoglobulin G (IgG) levels, lower complement 3 levels, and reduced white blood cell and hemoglobin levels. Logistic regression analysis identified lymphadenopathy as a risk factor and arthritis as a protective factor for the development of secondary ITP. The study reveals distinct clinical and hematological characteristics in pSS patients with secondary ITP, suggesting a higher disease activity in this subset. These findings underscore the need for further exploration of these associations to develop more precise treatment approaches for pSS, focusing on preventing secondary ITP and improving patient outcomes.

## 1. Introduction

Sjögren syndrome (SS) is an autoimmune disorder characterized by the infiltration of lymphocytes into the exocrine glands, primarily affecting the salivary and lacrimal glands. This condition manifests predominantly as xerostomia (dry mouth) and keratoconjunctivitis sicca (dry eyes), leading to significant morbidity.^[1]^ SS etiology involves a complex interplay of genetic, environmental, and immunological factors. SS is classified into primary (pSS) and secondary forms, the former occurring in the absence of any other autoimmune disease, while the latter is associated with other autoimmune conditions such as rheumatoid arthritis or systemic lupus erythematosus.^[2,3]^ immune thrombocytopenia (ITP) is an autoimmune condition marked by reduced platelet counts and an increased risk of bleeding. ITP pathogenesis is multifactorial, involving platelet destruction mediated by autoantibodies and impaired platelet production. ITP can be categorized as primary (idiopathic) or secondary, where it is associated with other diseases, including autoimmune disorders like SS.^[4,5]^

The intersection of pSS and secondary ITP presents a unique clinical challenge. Although the association between pSS and secondary ITP is recognized, the pathophysiological mechanisms underlying this relationship remain poorly understood.^[6]^ The variable clinical presentations of pSS, ranging from mild glandular dysfunction to systemic manifestations involving multiple organ systems, further compound this gap in knowledge. Clinical features of pSS-associated secondary ITP include, but are not limited to, mucocutaneous bleeding, petechiae, and menorrhagia. The diagnosis is often challenging, requiring careful exclusion of other causes of thrombocytopenia. Hematological parameters, such as platelet count and mean platelet volume, along with autoantibody profiles, provide essential insights into the disease state.^[7,8]^ However, a comprehensive analysis encompassing the full spectrum of clinical and hematological parameters in pSS patients with secondary ITP is still lacking.

The primary objective of this study is to fill this knowledge gap by conducting an in-depth analysis of the clinical manifestations and hematological parameters associated with secondary ITP in patients with pSS. This investigation aims to elucidate the extent of the impact of secondary ITP on pSS patients and to explore potential pathophysiological mechanisms that may contribute to this association. By doing so, this research endeavors to enhance the understanding of pSS as a systemic disease with multifaceted hematological implications and to facilitate more informed, targeted therapeutic strategies for managing patients with this complex comorbidity.

## 2. Methods

### 2.1. Study design

This investigation undertook a comprehensive retrospective analysis at our institution, aiming to delineate the clinical manifestations and hematological parameters associated with secondary ITP in patients diagnosed with pSS. The period of this retrospective inquiry spanned from January 2020 to December 2023. A total of 107 patients were included in the study, stratified into 2 distinct groups based on the occurrence of ITP during their disease course. The first group encompassed 21 patients who developed secondary ITP (the secondary ITP group), while the second comprised 86 patients who did not exhibit ITP (the Non-secondary ITP group). This segmentation allowed for a comparative analysis to ascertain the specific clinical and hematological attributes attributable to the onset of secondary ITP in the context of pSS. In compliance with ethical research practices, every participant involved in this study provided informed consent. The consent process entailed a thorough explanation of the study's purpose, methods, and potential implications for patient care. Ensuring the ethical integrity of our research, the study design, methodology, objectives, and protocols underwent a rigorous review and received subsequent approval from the Ethics Committee of our institution.

### 2.2. Inclusion and exclusion criteria

#### 2.2.1. Inclusion criteria

1. Diagnosis of pSS: Patients must have a formal diagnosis of pSS, as defined by the American-European Consensus Group criteria or the American College of Rheumatology/European League Against Rheumatism (ACR/EULAR) classification criteria.
2. Age and Gender: Participants of any gender aged 18 years or older.
3. Hematological Data: Availability of complete hematological profiles, including but not limited to platelet counts, mean platelet volume, and other relevant parameters throughout the course of pSS.

#### 2.2.2. Exclusion criteria

1. Other Autoimmune or Hematological Disorders: Patients with co-existing autoimmune diseases (other than pSS) or primary hematological disorders that could independently affect platelet counts or mimic ITP.
2. Prior Splenectomy: Patients who have undergone splenectomy, as this procedure significantly alters the hematological profile and could confound the study results.
3. Recent Blood Transfusions: Patients who received blood transfusions within 3 months prior to the start of the study period, due to the potential impact on hematological parameters.
4. Lack of Informed Consent: Patients who did not provide or were unable to provide informed consent for their medical data to be used in the study.

### 2.3. Multi-modal assessment strategy for evaluating primary Sjögren syndrome

In this study, a comprehensive approach was adopted to evaluate patients with pSS using a suite of established assessment tools. The European League Against Rheumatism Sjögren Syndrome Disease Activity Index (ESSDAI)^[9]^ was utilized to quantitatively measure the disease activity in pSS patients. Patient-reported symptoms were assessed using the EULAR Sjögren Syndrome Patient-Reported Index (ESSPRI),^[10]^ which focuses on subjective symptoms like pain, fatigue, and dryness. The Health Assessment Questionnaire (HAQ)^[11]^ was employed to gauge the patients’ ability to perform daily activities, thus providing insight into physical functioning and disability. Fatigue symptoms were quantified using the Fatigue Visual Analogue Score,^[12]^ with a score >1 indicating significant fatigue, while a score of 1 or less signified the absence of fatigue. Lymph node enlargement was defined as nodes exceeding 1 cm in length as detected by ultrasonography or high-resolution computed tomography (CT), or a reduction in the long-to-short axis ratio. Additionally, tear production was evaluated through the Schirmer test,^[13]^ conducted by ophthalmologists, with the lowest value between the 2 eyes being recorded for analysis.

### 2.4. Data collection and variables examined

This study involved comprehensive data collection to identify factors potentially influencing surgical outcomes. The information gathered encompassed a wide array of patient data, including initial clinical presentations upon hospital admission, laboratory test results, radiological imaging findings, and disease severity scores. For parameters that were assessed multiple times, the first set of results obtained after hospital admission was selected for analysis. This approach ensured a standardized baseline for comparison across all patients. All laboratory and diagnostic evaluations were completed within 72 hours of admission, providing a consistent and timely assessment of each patient condition. The data collection process was conducted in strict adherence to ethical standards.

### 2.5. Statistical analysis

In this investigation, statistical analyses were meticulously performed using SPSS software (Version 27.0). Data were first classified into quantitative or categorical categories, with normality tests applied to ascertain their distribution patterns. Quantitative data that conformed to a normal distribution were analyzed for inter-group statistical significance using independent sample *t* tests, and results were presented as mean ± standard deviation. For quantitative data not following a normal distribution, median and interquartile ranges (M[P25, P75]) were used for representation, and the Mann–Whitney U test was employed for comparing between groups. Categorical data were expressed as frequencies and percentages, and the independence or associations among these variables were analyzed using Chi-square (*χ*^2^) tests. Correlation analyses were conducted using Pearson correlation for normally distributed quantitative data, Spearman rank correlation for nonnormally distributed quantitative data, and Kendall tau for count data. Additionally, binary logistic regression analysis was utilized to identify potential risk factors contributing to the development of ITP in patients with pSS. This method allowed for an in-depth examination of various variables and their potential impact on the risk of developing ITP. All statistical tests in this study were 2-tailed, and a *P* value of <.05 was established as the threshold for statistical significance.

## 3. Results

### 3.1. Clinical and demographic characteristics in primary Sjögren syndrome with and without secondary immune thrombocytopenia

In the investigation of pSS patients, significant differences emerged when comparing those with secondary ITP to those without. Notably, the prevalence of fatigue was significantly higher in the secondary ITP group (90.5%) compared to the non-secondary ITP group (48.8%, *P* < .05). Similarly, a higher incidence of lymphadenopathy (57.1% vs 50%, *P* < .05) and arthritis (38.1% vs 59.3%, *P* < .05) was observed in the secondary ITP cohort. Moreover, the secondary ITP group presented with a lower mean age (55.5 years vs 59.9 years, *P* < .05) and a higher mean ESSDAI score (6.0 vs 5.0, *P* < .05), suggesting a greater disease activity. Conversely, the Schirmer test results indicated more severe ocular dryness in the non-secondary ITP group (5.0 mm/5 min vs 2.5 mm/5 min, *P* < .05). No statistically significant differences were found in renal tubular acidosis, neurological damage, interstitial lung disease, oral dryness, or HAQ scores between the groups (Table 1).

**Table 1:** Clinical characteristics comparison between secondary ITP and non-secondary ITP groups in primary Sjögren syndrome patients.

Parameter/Group	Secondary ITP group (n = 21)	Non-secondary ITP group (n = 86)	t/Z/*χ*^2^ value	*P* value
Renal tubular acidosis, n (%)	1 (4.8)	1 (1.2)	0.015	>.05
Neurological damage, n (%)	1 (4.8)	2 (2.3)	0.015	>.05
Interstitial lung disease, n (%)	2 (9.5)	12 (14.0)	0.033	>.05
Ocular dryness, n (%)	15 (71.4)	50 (58.1)	0.057	>.05
Oral dryness, n (%)	17 (81.0)	60 (69.8)	0.061	>.05
Dental caries, n (%)	6 (28.6)	17 (19.8)	0.246	>.05
Female, n (%)	20 (95.2)	77 (89.5)	0.497	>.05
Sialadenitis, n (%)	2 (9.5)	9 (10.5)	0.497	>.05
Proteinuria, n (%)	1 (4.8)	1 (1.2)	0.528	>.05
Schirmer test (mm/5 min)	2.5 (1.0, 4.0)	5.0 (±3.0)	2.465	<.05
Arthritis, n (%)	8 (38.1)	51 (59.3)	5.166	<.05
Lymphadenopathy, n (%)	12 (57.1)	43 (50.0)	6.878	<.05
Fatigue, n (%)	19 (90.5)	42 (48.8)	7.592	<.05
Age (yr)	55.5 (±11.0)	59.9 (±13.2)	−2.026	<.05
ESSDAI score (points)	6.0 (±2.8)	5.0 (±1.7)	−2.025	<.05
ESSPRI score (points)	24.0 (±7.0)	22.0 (±7.0)	−1.229	>.05
HAQ score (points)	5.0 (±3.0)	4.5 (±2.7)	−1.096	>.05
Disease duration (yr)	2.2 (1.0, 3.0)	2.2 (0.3, 5.0)	−0.074	>.05

ESSDAI = European League Against Rheumatism Sjögren Syndrome Disease Activity Index, ESSPRI = European League Against Rheumatism Sjögren Syndrome Patient-Reported Index, HAQ = Health Assessment Questionnaire, ITP = immune thrombocytopenia.

### 3.2. Hematological and immunological profiles in primary Sjögren syndrome with secondary immune thrombocytopenia

In a cohort of patients with primary Sjögren syndrome, those with secondary immune thrombocytopenia exhibited significantly different hematological and immunological parameters compared to those without secondary ITP. Notably, the platelet count was substantially higher in the secondary ITP group (184.1 ± 51.1 × 10^9^/L) versus the non-secondary ITP group (69.5 ± 18.2 × 10^9^/L, *P* < .01). Furthermore, Lymphopenia was more prevalent in the secondary ITP group (66.7% compared to 30.2%, *P* < .05), as was the mean concentration of immunoglobulin G (IgG) (17.0 ± 5.7 g/L vs 14.8 ± 4.9 g/L, *P* < .05). Levels of complement 3 were lower in the secondary ITP group (0.81 ± 0.27 g/L), indicating a potential association with disease activity (*P* < .05). White Blood Cell counts were also lower in patients with secondary ITP (3.4 ± 1.3 × 10^9^/L, *P* < .01), and a lower mean hemoglobin level suggested a trend toward anemia in this group (104.0 ± 20.2 g/L, *P* < .05). No significant differences were observed in IgA levels, prevalence of anemia, and positive rates of Anti-SSB, Rheumatoid Factor, Anti-SSA, and Anti-Ro52 Antibodies between the 2 groups (Table 2).

**Table 2:** Serum hematological parameters in primary Sjögren syndrome patients with and without secondary immune thrombocytopenia.

Parameter	Secondary ITP group (n = 21)	Non-secondary ITP group (n = 86)	t/Z value	*P* value
Platelet count (×10^9^/L)	184.1 ± 51.1	69.5 ± 18.2	16.54	<.01
Lymphopenia, n (%)	14 (66.7)	26 (30.2)	6.17	<.05
IgG (g/L)	17.0 ± 5.7	14.8 ± 4.9	−1.96	<.05
Complement 3 (g/L)	0.81 ± 0.27	0.99 ± 0.27	−2.1	<.05
WBC count (×10^9^/L)	3.4 ± 1.3	4.3 ± 1.7	−2.38	<.01
Hemoglobin (g/L)	104.0 ± 20.2	110.7 ± 14.9	−2.095	<.05
IgA (g/L)	2.7 ± 0.45	2.8 ± 1.08	−0.96	>.05
Anemia, n (%)	12 (57.1)	25 (29.1)	2.56	>.05
Anti-SSB antibody positive, n (%)	11 (52.4)	29 (33.7)	1.42	>.05
Rheumatoid factor positive, n (%)	16 (76.2)	47 (54.7)	0.66	>.05
Anti-SSA antibody positive, n (%)	18 (85.7)	78 (90.7)	0.47	>.05
Anti-Ro52 antibody positive, n (%)	17 (81.0)	68 (79.1)	0.05	>.05
ESR (mm/h)	22.1 (9.9, 35.4)	26.1 (13.5, 43.2)	−0.59	>.05
IgM (g/L)	0.99 (0.63, 2.43)	1.08 (0.72, 1.35)	−0.64	>.05
Complement 4 (g/L)	0.18 (0.08, 0.27)	0.27 (0.18, 0.27)	−0.65	>.05
CRP (mg/L)	2.4 (1.2, 8.7)	1.7 (0.7, 5.6)	−1.55	>.05

CRP = C-reactive protein, ESR = erythrocyte sedimentation rate, Ig = immunoglobulin, ITP = immune thrombocytopenia, WBC = white blood cell.

### 3.3. Identifying predictive factors for secondary immune thrombocytopenia in primary Sjögren syndrome

The logistic regression analysis aimed to identify clinical predictors for the development of secondary ITP among pSS patients. The study revealed that lymphadenopathy was associated with an increased risk of secondary ITP (odds ratio (OR) 3.651, 95% confidence interval (CI) 1.032–11.366, *P* < .05), indicating a statistically significant relationship. Conversely, the presence of arthritis appeared to be a protective factor, associated with a reduced risk of developing secondary ITP (OR 0.295, 95% CI 0.082–0.698, *P* < .05). Other factors such as gender, elevated levels of IgG, decreased complement 3, reduced WBC count, and a positive Schirmer test did not reach statistical significance, suggesting no clear predictive value in this cohort for the development of secondary ITP (Table 3).

**Table 3:** Logistic regression analysis of factors associated with secondary immune thrombocytopenia in patients with primary Sjögren syndrome.

Factor	Odds ratio (OR)	95% CI	*P* value
Fatigue	0.345	0.028 to 3.471	>.05
Elevated IgG	1.413	0.369 to 4.183	>.05
Decreased complement 3	1.496	0.369 to 5.240	>.05
Decreased WBC	2.317	0.681 to 6.869	>.05
Positive Schirmer test	2.672	0.671 to 9.128	>.05
Lymphadenopathy	3.651	1.032 to 11.366	<.05
Arthritis	0.295	0.082 to 0.698	<.05

IgG = immunoglobulin G, WBC = white blood cell.

## 4. Discussion

Our findings elucidate the clinical and hematological nuances of secondary ITP within the pSS context, challenging traditional perceptions by revealing higher platelet counts in some patients. This anomaly suggests alternative pathophysiological pathways, possibly linked to compensatory thrombopoiesis or the impact of immunosuppressive therapies, emphasizing the need for tailored patient management. The correlation of secondary ITP with lymphadenopathy and its inverse relationship with arthritis offers new insights into pSS immune complexity, hinting at potential research avenues for targeted treatments. Utilizing comprehensive assessment tools like ESSDAI and ESSPRI is pivotal in detecting and managing secondary ITP, underscoring a holistic approach to pSS care that prioritizes personalized treatment plans. This study advocates for continued exploration into the dynamic interplay between pSS and secondary ITP, aiming to refine therapeutic strategies through longitudinal research and evidence-based practice adjustments.

pSS is an autoimmune disorder predominantly affecting exocrine glands, yet its spectrum extends beyond glandular involvement, manifesting in various extraglandular conditions, including ITP.^[14,15]^ ITP in the context of pSS poses significant clinical challenges, notably due to the risks of severe bleeding and its substantial impact on patients’ quality of life.^[16,17]^ This study delves into the complex interplay between pSS and secondary ITP, highlighting key differences in clinical presentations, demographic profiles, and both hematological and immunological parameters between pSS patients with and without secondary ITP.^[18,19]^ Our findings reveal a unique pattern in pSS patients with secondary ITP, characterized by increased fatigue, a higher incidence of lymphadenopathy and arthritis, and distinct hematological and immunological markers. These differences underscore the need for a nuanced understanding of pSS, emphasizing the importance of comprehensive clinical evaluation in patients with this condition. This study contributes to the growing body of knowledge in rheumatology and hematology, providing valuable insights into the multifaceted nature of pSS and its association with secondary hematologic complications like ITP.

The significantly higher prevalence of fatigue in the secondary ITP group may be due to several factors.^[20,21]^ Fatigue in autoimmune disorders is multifactorial, often associated with chronic inflammation, immune dysregulation, and cytokine dysregulation. It possible that the immune system persistent activation in secondary ITP contributes to the systemic energy depletion manifesting as fatigue.^[22,23]^ Additionally, fatigue could be a clinical indicator of more aggressive disease activity, as supported by the higher mean ESSDAI scores observed in the secondary ITP group.^[24,25]^ Lymphadenopathy association with secondary ITP could be reflective of generalized immune activation, with the lymphatic system being a central player in the immune response.^[26]^ The fact that lymphadenopathy is a risk factor for secondary ITP suggests that the same underlying immune dysregulation contributing to glandular inflammation in pSS may also predispose patients to hematological manifestations, such as ITP. The inverse relationship between arthritis and secondary ITP is intriguing. Arthritis may indicate a different immunological pathway within pSS that does not predispose to the development of secondary ITP.^[27]^ This divergence could be due to the differential expression of cytokines or specific genetic markers that modulate the immune response differently in the joints compared to the hematopoietic system.

The higher platelet counts observed in the secondary ITP group, contrary to the expected thrombocytopenia associated with ITP, offer a notable insight into the disease complexity within the context of pSS. This phenomenon may be attributed to various factors such as the compensatory increase in thrombopoiesis due to immune-mediated destruction of platelets, rebound thrombocytosis following immunosuppressive treatment, or the influence of glucocorticoids and other treatments on platelet levels. Additionally, the diagnostic criteria for secondary ITP and the timing of hematological assessments relative to treatment could have influenced these findings. This unexpected result emphasizes the intricate interplay between pSS and secondary ITP, necessitating a more detailed approach in diagnosing and monitoring these patients. Future studies should focus on longitudinal analyses to elucidate the mechanisms behind these hematological variations, contributing to a deeper understanding of pSS-associated secondary ITP. This aligns with our study findings on the clinical and hematological distinctions in pSS patients with secondary ITP, reinforcing the importance of comprehensive evaluation in this patient population.

The absence of a significant association between gender, elevated IgG, decreased complement 3, reduced WBC count, and the development of secondary ITP suggests that these factors alone do not confer a substantial risk.^[28]^ However, the logistic regression analysis indicates that lymphadenopathy increases the risk while arthritis appears protective. This dichotomy highlights the heterogeneous nature of pSS and the multifactorial etiology of secondary ITP within this disease spectrum.^[29]^ The lack of significant predictive value for a positive Schirmer test and other serological markers (Anti-SSA, Anti-SSB, Rheumatoid Factor, Anti-Ro52 Antibodies) suggests that secondary ITP development may not be directly mediated by these factors or that their roles may be overshadowed by more potent disease drivers.

This study, while providing valuable insights, has several limitations. Firstly, its retrospective design may limit the ability to establish causality between pSS and secondary ITP. Secondly, the sample size, particularly of the secondary ITP group, is relatively small, potentially impacting the statistical power and generalizability of the findings. Additionally, the study focuses on a specific patient population, which might limit its applicability to broader demographic groups. Lastly, the reliance on clinical and laboratory records may introduce bias, as the data quality and completeness depend on the accuracy of these records.

Building on our findings, future research should delve deeper into pSS and secondary ITP interrelations. Studies are essential to monitor hematological parameter shifts in pSS, especially under various treatments, to uncover pathophysiological mechanisms and potential biomarkers for secondary ITP. Exploring the effectiveness and outcomes of specific immunosuppressive treatments will also be critical in developing targeted therapies. Additionally, investigating the genetic and molecular bases of pSS and secondary ITP may reveal new intervention targets, promoting personalized treatment approaches. Integrating rheumatology, hematology, and immunology through multidisciplinary studies can enhance our understanding and management of this condition, ultimately improving patient care. Addressing these research avenues will significantly advance our grasp of pSS and its hematologic complications, paving the way for more effective management strategies.

## 5. Conclusions

In conclusion, this study has unveiled unique clinical characteristics of pSS patients with secondary ITP, highlighting a higher disease activity in this group. These findings provide pivotal evidence contributing to a deeper understanding of the disease. By further exploring these associations, we can advance toward more nuanced treatment approaches for pSS, aiming ultimately to prevent the development of secondary ITP and enhance the quality of life for affected patients.

## Author contributions

**Conceptualization:** Wenwen Yang.

**Data curation:** Wenwen Yang.

**Formal analysis:** Wenwen Yang.

**Investigation:** Wenwen Yang.

**Methodology:** Wenwen Yang.

**Resources:** Wenwen Yang.

**Software:** Wenwen Yang.

**Writing – original draft:** Wenwen Yang.

**Writing – review & editing:** Wenwen Yang.
